# Targeting Toll-Like Receptors: Promising Therapeutic Strategies for the Management of Sepsis-Associated Pathology and Infectious Diseases

**DOI:** 10.3389/fimmu.2013.00387

**Published:** 2013-11-18

**Authors:** Athina Savva, Thierry Roger

**Affiliations:** ^1^Infectious Diseases Service, Department of Medicine, Centre Hospitalier Universitaire Vaudois, University of Lausanne, Lausanne, Switzerland

**Keywords:** Toll-like receptor, innate immunity, cytokine, sepsis, infectious disease, clinical trial, therapy

## Abstract

Toll-like receptors (TLRs) are pattern recognition receptors playing a fundamental role in sensing microbial invasion and initiating innate and adaptive immune responses. TLRs are also triggered by danger signals released by injured or stressed cells during sepsis. Here we focus on studies developing TLR agonists and antagonists for the treatment of infectious diseases and sepsis. Positioned at the cell surface, TLR4 is essential for sensing lipopolysaccharide of Gram-negative bacteria, TLR2 is involved in the recognition of a large panel of microbial ligands, while TLR5 recognizes flagellin. Endosomal TLR3, TLR7, TLR8, TLR9 are specialized in the sensing of nucleic acids produced notably during viral infections. TLR4 and TLR2 are favorite targets for developing anti-sepsis drugs, and antagonistic compounds have shown efficient protection from septic shock in pre-clinical models. Results from clinical trials evaluating anti-TLR4 and anti-TLR2 approaches are presented, discussing the challenges of study design in sepsis and future exploitation of these agents in infectious diseases. We also report results from studies suggesting that the TLR5 agonist flagellin may protect from infections of the gastrointestinal tract and that agonists of endosomal TLRs are very promising for treating chronic viral infections. Altogether, TLR-targeted therapies have a strong potential for prevention and intervention in infectious diseases, notably sepsis.

## Introduction

Sepsis is one of the leading causes of death worldwide. Incidence of severe sepsis is increasing and mortality rates remain significantly high despite early care management (1). Moreover, more than 30% of survivors develop long-term functional disabilities and cognitive impairments (2). The Surviving Sepsis Campaign is a global initiative incepted in early 2000s with the aim to improve sepsis diagnosis and treatment in order to enhance the awareness of sequelae and to decrease high mortality rates associated with sepsis^1^. In collaboration with many countries in Europe and the United States, the Surviving Sepsis Campaign suggests evidence-based guidelines and bundles. The most recent guidelines recommend acute resuscitation of septic patients, administration of antibiotics and support of organ failure. Yet, no treatment targeting the underlying mechanism of sepsis is actually available (3). Recombinant human activated protein C (rhAPC, Xigris^®^, Eli Lilly), the only drug specifically registered for sepsis, has recently been withdrawn from the market following the negative results from the PROWESS-SHOCK study that did not show reduction in mortality at 28 or 90 days in patients with septic shock (4).

It is generally admitted that sepsis results from a dysregulated host response to an initial insult, characterized by inflammation mediating tissue damage and organ failure and an immune suppression state responsible for the development of secondary infections (5–7). The immune response to an infection is initiated by the sensing of microbial structures through families of receptors collectively called pattern recognition receptors (PRRs). The most well-described families comprise Toll-like receptors (TLRs), nucleotide binding oligomerization domains (NODs)-like receptors (NLRs), c-type lectin receptors (CLRs, such as dectin-1, dectin-2, DC-SIGN), RIG-I-like receptors (RLRs, RIG-I, and MDA5), and intra-cytosolic DNA sensors. PRRs are expressed by innate immune cells like dendritic cells and macrophages. The binding of microbial ligands to PRRs promotes the release of mediators, among which cytokines, that initiate and regulate the inflammatory response necessary to eliminate invasive pathogens and coordinate the development of the adaptive immune response (8, 9). Innate immune cells are also triggered by damage (or danger)-associated molecular patterns (DAMPs), known as alarmins. DAMPs are endogenous components commonly released by injured or stressed cells, such as nucleic acids, histones, uric acid crystals, ATP, cytochrome c, S100 molecules, and HMGB1. DAMPs are primarily sensed through the NLRP3 inflammasome, which controls the secretion of IL-1β and IL-18 (10).

The concept of PRRs sensing microbial-associated molecular patterns (MAMPs) and discriminating self from non-self molecular structures was proposed by Janeway (11). Two major cornerstone discoveries largely confirmed Janeway’s concept. The first one was the demonstration of the essential antifungal role of the Toll protein in *Drosophila* (12). The second one arose from the positional cloning linking LPS (commonly called endotoxin) unresponsive phenotype of C3H/HeJ and C57BL/10ScCr strains of mice to missense and null mutations of the *Toll-like receptor 4* (*Tlr4*) gene (13). The importance of these discoveries and of the role of dendritic cells as central regulators of innate and adaptive immunity has been acknowledged by the 2011 Nobel Prize in Physiology or Medicine attributed to Bruce A. Beutler and Jules A. Hoffmann “for their discoveries concerning the activation of innate immunity” and to Ralph M. Steinman “for his discovery of the dendritic cell and its role in adaptive immunity” (14).

## Toll-Like Receptors

Toll-like receptors belong to the most studied family of PRRs, due to their central role in host defenses and involvement in a number of pathological processes that include sepsis. TLRs are type I trans-membrane proteins composed of an extracellular leucine-rich repeat (LRR) domain involved in ligand recognition, a trans-membrane domain, and a Toll-interleukin 1 receptor (TIR) domain involved in signaling (9, 15). About 10 functional human TLRs (TLR1-10) and 12 functional mouse TLRs (TLR1-9, TLR11-13) have been described, each one being involved in the sensing of distinct microbial products (Table 1). Expressed at the cell surface, TLR4 detects LPS from Gram-negative bacteria. TLR4 shuttles to late endosome to induce alternative signaling following LPS sensing. TLR2 as heterodimers in association with either TLR1 or TLR6 (and possibly TLR10) senses a variety of microbial products, such as lipopeptides, lipoproteins, peptidoglycan, porins, β-glucan, glycosylphosphatidylinositol (GPI) anchors, and glycoproteins from Gram-positive bacteria, Gram-negative bacteria, mycoplasma, mycobacteria, fungi, parasites, and viruses. TLR5 senses flagellin of bacterial flagella. TLR3, TLR7, TLR8, and TLR9 are strategically expressed in endosomal compartments to recognize microbial nucleic acids: double-stranded RNA (dsRNA) by TLR3, single-stranded RNA (ssRNA) by TLR7 and TLR8, and unmethylated CpG motif containing DNA by TLR9 (Table 1). It is therefore not surprising that endosomal TLRs have been primarily involved in host defenses against viruses, whereas TLR1, TLR2, and TLR4-6 have been mainly involved in host response to bacterial and fungal infection. TLRs cooperate with other molecules to recognize microbial ligands. For example, TLR2 requires CD14, CD36, and dectin-1 for the recognition of peptidoglycan, lipopeptides, and β-glucan, respectively. Of note, TLRs are also triggered by DAMPs released by injured or stressed cells during infection (16) (Table 1). TLR activation enables pathogen elimination by promoting bactericidal activity of leukocytes, and maturation and function of antigen presenting cells, thus orchestrating the development of adaptive immune responses (17).

**Table 1:** **Toll-like receptors and their ligands**.

Receptor	Sub-cellular expression	Adaptor molecule	Ligand	Origin
TLR1 (with TLR2)	Cell membrane	MyD88/TIRAP	Triacyl lipopeptides	Bacteria, mycobacteria
			Soluble factors	*Neisseria meningitidis*
TLR2 (with TLR1 or TLR6)	Cell membrane	MyD88/TIRAP	Lipoproteins, lipopeptides	Various pathogens
			Lipoteichoic acid	Gram-positive bacteria
			Peptidoglycan	Bacteria
			Lipoarabinomannan	Mycobacteria
			Phenol-soluble modulin, porins	*Staphylococcus epidermidis, Neisseria*
			Atypical LPS	*Leptospira interrogans, Porphyromonas gingivalis*
			Glycoinositolphospholipids, glycolipids	*Trypanozoma, Toxoplasma, Plasmodium*
			Beta-glucan, mannan	Fungi
			Core and NS3 proteins, dUTPase, glycoproteins	Hepatitis virus, Epstein–Barr virus, Cytomegalovirus
			HSP70	Host
TLR3	Endolysosomal	TRIF	Double-stranded RNA	Viruses
TLR4	Cell membrane and endolysosomal	MyD88/TIRAP, TRIF/TRAM	LPS	Gram-negative bacteria
			O-linked mannan	Fungi
			Taxol	Plants
			Fusion and envelope protein	Respiratory syncytial virus, mouse mammary tumor virus
			HSP60	*Chlamydia pneumoniae*
			HMGB1, HSP70, fibronectin, fibrinogen	Host
TLR5	Cell membrane	MyD88	Flagellin	Flagellated bacteria
TLR6 (with TLR2)	Cell membrane	MyD88/TIRAP	Diacyl lipopeptides, lipoteichoic acid, β-glucan	*Mycoplasma*, Gram-positive bacteria, fungi
TLR7	Endolysosomal	MyD88	Single-stranded RNA	Viruses, bacteria
			Imidazoquinoline, loxoribine, bropirimine	Synthetic compounds
TLR8	Endolysosomal	MyD88	Single-stranded RNA	Viruses, bacteria
			Imidazoquinoline	Synthetic compounds
TLR9	Endolysosomal	MyD88	CpG-containing DNA	Bacteria, viruses, fungi
			Homozoin	*Plasmodium falciparum*
TLR10 (±TLR1 or TLR2)	Cell membrane	MyD88	Lipopeptides (prediction)	
TLR11	Endolysosomal	MyD88	Flagellin	Flagellated bacteria
TLR12	Endolysosomal	MyD88	Profilin	Apicomplexan parasites
TLR13	Endolysosomal	MyD88	23S RNA	Bacteria

The signaling pathways resulting from TLR triggering engage adaptors that are recruited by TIR/TIR domain interactions (Table 1): myeloid differentiation primary response gene (18) (MyD88), TIR domain-containing adaptor protein (TIRAP, also known as MAL), TIR domain-containing adaptor inducing interferon (IFN)β (TRIF), and TRIF related adaptor molecule (TRAM) (19). MyD88 is essential for signaling through all TLRs except TLR3 and is involved in early nuclear factor-κB (NF-κB) and mitogen-activated protein kinases (MAPKs) activation and pro-inflammatory gene expression. TIRAP serves as a bridge to recruit MyD88 to TLR2 and TLR4. TRIF initiates MyD88-independent IFN regulatory factor 3 (IRF3) and late NF-κB activation involved in the production of type I IFNs and IFN-inducible genes (19, 20). TRIF is recruited to the cytoplasmic domain of TLR3 and, in late endosome, through TRAM that bridges TRIF to TLR4. A fifth TIR domain-containing adaptor, sterile α-, and armadillo-motif containing protein (SARM) acts as a negative regulator of TLR3 and TLR4 signaling. SARM interacts with TRIF and inhibits the induction TRIF-dependent genes.

The signaling pathways activated downstream TLRs have some redundancy. Yet, the engagement of multiple TLRs, especially MyD88 and TRIF-dependent TLRs, have synergistic effects on host responses (21, 22). Intracellular cross talk between signaling pathways may also occur when different families of PRRs are involved. For example, dectin-1 synergizes with TLR2 and TLR4 and increases cytokine production through canonical and non-canonical NF-κB pathways (23, 24). Moreover, a single MAMP can be detected by different PRRs. This differential sensing is primarily depending on the localization of the MAMP. Indeed, flagellin is sensed by TLR5 expressed at the cell surface and by the NAIP5/NLRC4 (also known as IPAF) inflammasome when localized in the cytoplasm (25–27). Similarly, peptidoglycans stimulate membrane TLR2 and intra-cytosolic NOD1/NOD2-dependent cell activation (28). Interestingly, some CpG and non-CpG oligodeoxynucleotides directly stimulate and polarize T-cells through TLR9 and MyD88-independent mechanisms (29), possibly through intracellular DNA sensors. Recently, Hagan et al. and Kayagaki et al. demonstrated non-canonical TLR4-independent recognition of intracellular LPS through an uncharacterized receptor (30, 31). This unconventional mode of LPS sensing activates caspase-11-dependent IL-1β secretion and sensitizes mice to endotoxic shock. All these observations indicate that the host has evolved different strategies to sense invading microorganisms. Ideally, all possible interactions should be characterized and/or anticipated, so that the effect of treatment application can be predicted and/or translated. This mandates carefully planned experiments that represent real-life conditions and a detailed knowledge of compound’s mode of action. Obviously, the redundancy of microbial sensing pathways should be taken into consideration when developing or applying targeted-treatment strategies to a single PRR.

Experimental animal models and human clinical studies support a crucial role for TLRs in infectious diseases. The first evidence came from the observation that TLR4 defective C3H/HeJ and C57BL/10ScCr mice are hyporesponsive to LPS and susceptible to otherwise non-lethal infection with *Escherichia coli* and *Salmonella typhimurium*. Subsequent studies with mice knockout in TLRs or TLR adaptor molecules have demonstrated the importance of the TLR pathway in host defenses. For example, TLR2 knockout mice are highly susceptible to infections by *Staphylococcus aureus* and *Streptococcus pneumoniae* (32). More recently, human association studies have linked polymorphisms affecting TLR expression or TLR structure with an augmented propensity to develop infections (32–35).

The discovery of TLRs and their involvement in innate immune responses has attracted much interest into the development of drugs for controlling infections and improving sepsis management. This field of research has been very dynamic, and numerous compounds focused on TLRs have been tested in animals and human subjects. TLR agonists are powerful adjuvant constituting the heart of vaccine efficacy enhancement. Moreover, they are promising TLR-directed agents developed for autoimmune diseases and cancer (36). These two particular aspects of the TLR-targeting field will not be addressed in this review. Herein, we will review the most popular agonist (TLR3, TLR5, TLR7, TLR8, TLR9) and antagonist (TLR2, TLR3, TLR4, TLR9) agents used in pre-clinical and clinical models of acute and chronic infections, including sepsis. Relevant registered clinical trials^2^ are listed in Table 2.

**Table 2:** **Selection of clinical trials testing drugs targeting TLRs for bacterial and viral infections**.

Compound	Company/organism	Target	Effect	Clinical phase	Clinical trial no.	Aim	Comment
E5564 (eritoran)	Eisai Inc	TLR4	Antagonist	Randomized, double-blind phase 2	NCT00046072	Safety and efficacy in patients with severe sepsis	(37)
E5564	Eisai Inc	TLR4	Antagonist	Randomized, double-blind phase 3	NCT00334828	Treatment and reduced mortality of severe sepsis	(38)
TAK-242 (Resatorvid)	Takeda	TLR4	Antagonist	Randomized, double-blind phase 3	NCT00143611	Efficacy and safety in patients with severe sepsis	(39)
TAK-242	Takeda	TLR4	Antagonist	Randomized, double-blind phase 3	NCT00633477	Efficacy and safety in patients with sepsis-induced cardiovascular and respiratory failure	Study terminated
NI-0101	NovImmune SA	TLR4	Antagonist	Randomized, double-blind phase 1	NCT01808469	Safety and PK/PD* in healthy volunteers	Currently recruiting
Poly-ICLC (Hiltonol)	Rockefeller University	TLR3	Agonist	Randomized, double-blind phase 1	NCT00646152	Safety and tolerability in healthy volunteers	Active, not recruiting
FluMist + Poly(I:C_12_U)	Hemispherx Biopharma	TLR3	Agonist	Randomized, double-blind phase 1 and phase 2	NCT01591473	Immunogenicity and safety of human influenza vaccine in healthy volunteers	Currently recruiting
Poly(I:C_12_U)	Hemispherx Biopharma	TLR3	Agonist	Randomized, open label phase 2	NCT00035893	Effectiveness in increasing the HAART-free time interval before HIV rebound	No results available
Poly(I:C_12_U)	Hemispherx Biopharma	TLR3	Agonist	Randomized, open label phase 2	NCT00035581	Safety and efficacy in combination with HAART in HIV subjects	Study terminated
SD-101	Dynavax Technologies Corporation	TLR9	Agonist	Randomized, single blind phase 1	NCT00599001	Safety and PK/PD in healthy male volunteers	No results available
SD-101	Dynavax Technologies Corporation	TLR9	Agonist	Randomized, single blind phase 1	NCT00823862	Efficacy and PK/PD in treatment-naïve genotype 1 HCV subjects	(40)
IMO-2125	Idera Pharmaceuticals, Inc	TLR9	Agonist	Randomized, double-blind phase 1	NCT00728936	Dose escalation and PK/PD in non-responders HCV subjects	(41)
IMO-2125	Idera Pharmaceuticals, Inc	TLR9	Agonist	Randomized, double-blind phase 1	NCT00990938	Dose escalation, safety, and PK/PD in treatment-naïve genotype 1 HCV subjects	(42)
CPG10101	Pfizer	TLR9	Agonist	Randomized, open label phase 1	NCT00142103	Treatment of relapsed HCV subjects	No results available
CPG10101	Pfizer	TLR9	Agonist	Randomized, open label phase 2	NCT00277238	Efficacy and PK/PD in non-responders HCV subjects	No results available
Chloroquine	Assistance Publique Hôpitaux De Marseille	TLR9 and TLR4	Unknown	Randomized, double-blind phase 3	NCT00391313	Efficacy and safety in patients with Chikungunya	Study terminated
Chloroquine	National University Hospital, Singapore	TLR9 and TLR4	Unknown	Randomized, double-blind phase 2	NCT01078779	Prevention of influenza	(43)
Chloroquine	University of Sao Paulo	TLR9 and TLR4	Unknown	Randomized, double-blind phase 1 and phase 2	NCT00849602	Treatment of patients with dengue	(44)
Imiquimod (Aldara)	Graceway Pharmaceuticals, LCC	TLR7	Agonist	Randomized, double-blind phase 3	NCT00735462 NCT00674739	Treatment, safety, and effectiveness in external genital warts due to HPV	(45)
Resiquimod (R-848)	Graceway Pharmaceuticals, LCC	TLR7 and TLR8	Agonist	Non-randomized, single blind phase 2	NCT00116662 NCT00116675	Safety and efficacy in common warts in pediatric subjects up to 4 and 12 weeks	No results available
Resiquimod	Graceway Pharmaceuticals, LCC	TLR7 and TLR8	Agonist	Non-randomized, single blind phase 2	NCT00114920 NCT00115141 NCT00117871 NCT00117923	Safety and efficacy in common warts in adults up to 4 and 12 weeks	No results available
Imiquimod (Aldara)	Medical University of Vienna	TLR7	Agonist	Non-randomized, single blind phase 2	NCT00941811	Treatment of vulvar intraepithelial neoplasias 2/3 (VIN) and ano-genital warts	Unknown
Miltefosine and Imiquimod	Foundation Fader	TLR7	Agonist	Randomized, double-blind phase 2	NCT01380314	Treatment of cutaneous leishmaniasis	No results available
Imiquimod	MEDA Pharma GmbH and Co. KG	TLR7	Agonist	Randomized, double-blind phase 4	NCT00189293	Recurrence rate after treatment of external ano-genital warts	No results available
Imiquimod	MEDA Pharma GmbH and Co. KG	TLR7	Agonist	Non-comparative, open label phase 4	NCT00761371	Treatment of external genital or perianal warts in HIV subjects	(46)
Antimony and Imiquinod	Drugs for Neglected Diseases	TLR7	Agonist	Randomized, double-blind phase 3	NCT00257530	Treatment of cutaneous leishmaniasis	(47)
Imiquimod	Assistance Publique-Hopitaux de Paris	TLR7	Agonist	Randomized, open label phase 3	NCT01059110	Treatment of plantar warts	Currently recruiting
Imiquimod	Conrad	TLR7	Agonist	Randomized, open label phase 2	NCT01593124	Immune response of vaginal tissue after exposure to product	Active, not recruiting
ANA 773	Hoffmann-La Roche	TLR7	Agonist	Randomized, double-blind phase 1	NCT01211626	Safety and PK/PD in HCV subjects and healthy volunteers	(48, 49)
PF-04878691	Pfizer	TLR7	Agonist	Randomized, double-blind phase 1	NCT00810758	Safety and PK/PD in healthy volunteers	(50)
GS-9620	Gilead Sciences	TLR7	Agonist	Randomized, double-blind phase 1	NCT01591668	Safety and PK/PD in treatment-naïve HCV subjects	Active, not recruiting
GS-9620	Gilead Sciences	TLR7	Agonist	Randomized, double-blind phase 1	NCT01590641 NCT01590654	Safety, PK/PD in HBV virologically suppressed subjects and in treatment-naïve HBV subjects	Currently recruiting

*PK/PD: Pharmacokinetics/Pharmacodynamics.

## Toll-Like Receptor 4 Antagonists

LPS is the main pro-inflammatory molecule anchored in the outermembrane of Gram-negative bacteria (51). Neutralization of bacterial LPS, inhibition of its recognition by host cells or inhibition of signaling downstream LPS binding to its receptor has long been considered a promising approach for the treatment of severe sepsis and septic shock. Interestingly, endotoxemia is prevalent in septic patients, not only in those with Gram-negative infection. Indeed, translocation of viable bacteria and LPS from the gastrointestinal tract has been proposed to participate in the pathophysiology of sepsis. TLR4 was identified 15 years ago as the signal-transducing molecule of the LPS receptor complex (13), which also comprises MD-2 and CD14. Thus, TLR4 is regarded as a primary target for treating sepsis (52). TLR4 expression is increased in human monocytes of healthy volunteers challenged with LPS (53), as well as in patients with sepsis (54). Moreover, polymorphisms in the *TLR4* gene have been associated with Gram-negative sepsis (33, 35). In the following sections, we present the most advanced TLR4 antagonists developed for the treatment of sepsis.

### Eritoran-E5564

Strategies to inhibit LPS-mediated toxic effects have been initiated years before the discovery of TLR4 (13) and the unraveling of the crystal structure of the TLR4-MD-2-LPS complex (55). Lipid A, the toxic moiety of LPS, is highly conserved among endotoxins and constitutes an ideal therapeutic target (56). E5531, developed by Eisai Research Institute of Boston (Andover, MA, USA), was the first-generation lipid A antagonist derived from *Rhodobacter capsulatus* endotoxin. E5531 conferred protection in experimental models of endotoxemia and lethal infection with *E. coli* (57). The protective effect likely occurred through the binding of E5531 to the TLR4-MD-2 complex and the inhibition of the interaction between LPS and TLR4-MD-2 (58). E5531 also blocked endotoxin response in human healthy volunteers challenged intravenously with LPS (59). E5531 development went through phase 2 clinical trial, but was stopped due to issues of bioavailability.

A second-generation LPS antagonist drug candidate developed by Eisai is eritoran tetrasodium (known as eritoran or E5564), a synthetic lipid A analog of *Rhodobacter sphaeroides* (60). Eritoran blocked LPS-induced cytokines *in vitro* and in experimental animal models (61–63). In a phase 1 clinical trial enrolling healthy volunteers challenged with LPS, eritoran inhibited pro-inflammatory cytokine production and diminished clinical symptoms of sepsis, including fever, chills, tachycardia, and headache. Additionally, C-reactive protein levels and white blood counts were significantly decreased (64–67). The only adverse event observed was a dose-dependent phlebitis, due to the fact that high doses of eritoran were used to achieve stable activity of the drug over time.

A phase 2 randomized control trial recruiting critically ill septic patients as assessed by the Acute Physiology and Chronic Health Evaluation II (APACHE II) score disclosed a trend toward decreased mortality in the eritoran treated group (37). Phase 3 ACCESS (A Controlled Comparison of Eritoran and placebo in patients with Severe Sepsis) clinical trial for severe sepsis started in 2006, and results were published in 2013. About 1304 patients were treated with eritoran and 657 patients with placebo within 12 h after the onset of the first organ dysfunction. Unfortunately, analyses did not reveal reduced all-cause mortality in primary and secondary end-points (i.e., 28 days and 1 year mortality) (38). Eisai (Tokyo, Japan) waived to submit eritoran to marketing authorization for the treatment of severe sepsis in January 2011, based on preliminary results of the ACCESS trial.

Several reasons may account for the lack of efficacy of eritoran (68–70). For instance, patients were not enrolled or monitored based on the circulating levels of LPS, questioning about the appropriateness of inclusion criteria. It is also possible that eritoran would be more efficient if administrated rapidly, before septic shock is underway, pointing the early and aggressive sepsis management as a possible interfering factor. Other factors to take into account include the heterogeneity of patients for genetic background, underlying diseases, inflammatory and immune status, sepsis severity, infectious agent, and site of infection. As mentioned earlier, intracellular LPS sensed in a TLR4-independent manner sensitizes mice to endotoxic shock (30, 31). This non-canonical LPS detection may have limited the efficacy of the anti-TLR4 strategy. Moreover, upon infection, innate immune cells will likely sense several MAMPs via several TLRs and non-TLR PRRs. For example, Gram-negative bacteria express MAMPs that may trigger redundant inflammatory pathways through TLR2 (lipopeptides), TLR4 (LPS), TLR5 (flagellin), TLR7 (ssRNA), and TLR9 (bacterial DNA). All these observations suggest that blocking one single pathway may be insufficient to interfere with the deleterious cascade of events observed in sepsis. The positive side of the ACCESS trial failure was a rethink of the design of sepsis clinical trials (68–70). Clearly, a drug like eritoran should be tested in selected patients and treatment efficacy examined and adjusted according to predefined appropriate biomarkers (such as LPS blood levels and genetic polymorphisms affecting the TLR4 pathway). A rigorous approach combining the power of “omics” technologies would allow the selection of homogeneous cohorts and the follow-up of the response to treatment, both of which are mandatory for the successful development of anti-sepsis drugs.

### TAK-242

Another anti-sepsis agent that exhibited promising therapeutic properties is TAK-242 [Ethyl-(6R)-[*N*-(2-chloro-4-fluorophenyl) sulfamoyl] or resatorvid] from Takeda Pharmaceutical Company (Osaka, Japan). TAK-242 was originally characterized as a suppressor of nitric oxide (NO) and cytokine production by LPS-stimulated macrophages and during endotoxic shock in mice (71). TAK-242 binds to cysteine 747 in the intracellular domain of TLR4, thereby inhibiting both MyD88-dependent and MyD88-independent pathways activated by LPS (72). When administered in conscious guinea pigs following LPS challenge, TAK-242 significantly improved septic shock symptoms, decreasing HMGB1 systemic levels, and increasing survival in a dose-dependent manner (73). TAK-242 also increased survival rates from 17 to 50% and improved organ dysfunction when co-administered with antibiotics in a mouse model of cecal ligation and puncture (CLP). No effect on circulating bacterial counts was observed (74). A double-blind, randomized, placebo-controlled trial was initiated with TAK-242 (39). Inclusion criteria comprised symptoms of severe sepsis accompanied with either shock and/or respiratory failure. The study was stopped prematurely due to failure to achieve significant decrease of systemic cytokine levels at stage 1 of the analysis (39). A phase 3 clinical study was designed but never launched based on business decision and not due to safety or efficacy concerns.

### TLR4 antagonistic antibodies and other TLR4 targeting strategies

Antibodies directed against TLR4 or the TLR4-MD-2 complex have been generated and showed promising results in several pre-clinical studies. We engineered a soluble chimeric protein composed of the N-terminal and central domains of mouse TLR4 (amino acid 1–334) fused to the Fc domain of human IgG1 (75). The chimeric molecule was used to generate high titer anti-mouse TLR4 rabbit polyclonal antibodies. The anti-TLR4 antibodies powerfully inhibited NF-κB and MAPK activation and cytokine production by LPS-stimulated cells *in vitro*. The antibodies also hampered cytokine production and protected mice from lethal endotoxemia when administered both prophylactically and therapeutically 4 h after LPS. Prophylactic administration of anti-TLR4 antibodies blunted TNF production and strikingly increased survival in *E. coli* sepsis, from 0% in the control antibody group to 80% in the anti-TLR4 group. Even more impressive, anti-TLR4 therapy initiated as much as 13 h after the onset of infection in a model of *E. coli* peritoneal infection improved survival from 30 to 75% (75). Our studies demonstrate that anti-TLR4 antibodies are efficient as adjunctive therapy for *E. coli* sepsis, with a window of clinical application comprising prophylactic and therapeutic intervention opportunities. Several anti-TLR4 monoclonal antibodies have been produced. The group of Miyake (University of Tokyo, Japan) reported in 2000 the generation of MTS510, the first rat monoclonal antibody specific of the mouse TLR4-MD-2 complex (76). MTS510 was shown to inhibit LPS-induced NF-κB activation and TNF production by macrophages. 5E3 is a rat monoclonal antibody produced by NovImmune SA (Geneva, Switzerland) that reacts with the TLR4-MD-2 complex (77). 5E3 inhibited LPS-induced cell activation, and protected mice from lethal endotoxemia when injected up to 7 h after LPS challenge. Moreover, administration of 5E3 at the time of surgery improved the outcome of mice with colon ascendens stent peritonitis, a model of polymicrobial abdominal sepsis. Finally, the rat monoclonal antibody 1A6, that recognizes both mouse and human TLR4-MD-2 complexes, conferred protection in a model of *E. coli* sepsis, but not *Salmonella enterica*, sepsis (78).

Although some TLR4 inhibitors have entered clinical and pre-clinical trials, others remain in the developmental stage. LPS-Trap-Fc antibodies (comprising the extracellular domain of mouse TLR4 fused with MD-2 and linked to human IgG Fc) dose-dependently decreased IL-6 release by macrophages, opsonized Gram-negative bacteria, and enhanced phagocytosis and complement-mediated bacterial killing (79). Cell-penetrating peptides comprising the translocating segment of *Drosophila antennapedia* homeodomain fused with BB loop sequences of TLR4 (i.e., TLR4-BB peptides) inhibited LPS-induced NF-κB and MAPK activation and cytokine production (80, 81). Further studies will be required before advancing these products toward the clinical level.

Altogether, the experimental data reported above provided strong support for the concept of TLR4-targeted therapy for Gram-negative sepsis. In the gloomy context following the withdrawn of rhAPC and eritoran from the sepsis field, it is hopeful that NI-0101 has entered clinical development. NI-0101 is an anti-TLR4 monoclonal antibody produced by NovImmune able to block TLR4 dimerization and TLR4-mediated signaling triggered by LPS and endogenous and chemical ligands of TLR4. Data from pre-clinical studies in models of arthritis, respiratory inflammation, and organ injury have highlighted the potential favorable action of this agent^3^. A phase 1 clinical study is currently recruiting participants to evaluate drug safety and tolerance in healthy volunteers before and after *ex vivo* and *in vivo* LPS challenge. Pharmacokinetics and pharmacodynamics will also be assessed. Results from these studies are eagerly awaited.

### Targeting TLR4 in fungal and viral infection

Albeit less well characterized, TLR4 has been implicated in the sensing of non-bacterial microorganisms such as viruses and fungi. TLR4 recognizes O-linked mannan from *Candida albicans*, and human studies have linked Asp229Gly *TLR4* polymorphism with susceptibility to bloodstream candidiasis and pulmonary aspergillosis (82–84). In a model of disseminated infection with *C. albicans*, C3H/HeJ TLR4-deficient mice exhibited a 10-fold increased fungal load in the kidneys, which was associated with reduced production of the chemokines KC and MIP-2 and an impaired recruitment of neutrophils (85). Treatment with HTA 125, an anti-human TLR4 mouse monoclonal antibody (86), interfered with neutrophil-mediated protection against *C. albicans* invasion and cell injury in an *in vitro* epithelial model of oral candidiasis (87) and inhibited TNF production by human PBMCs stimulated with *Aspergillus* hyphae (88). It is still unclear whether targeting TLR4 may be beneficial in the context of fungal infections. A more clear yet unexpected picture has arisen from viral infection studies. Reactive oxygen species (ROS) produced by the NADPH oxidase generates oxidized host phospholipids that stimulate TLR4 and the production of cytokines involved in acute lung injury (89). Using a mouse model of lethal infection with influenza, the group of Stephanie Vogel (University of Maryland, Baltimore, MA, USA) reported that eritoran significantly increases survival in a dose-dependent manner even when administered 6 days after viral challenge. Lung pathology and clinical symptoms were improved while viral titers and influenza-induced cytokine gene expression in lung homogenates were decreased compared to the placebo-treated group. These data suggest that the therapeutic effect of eritoran in a more practical timing of severe sepsis treatment remains substantial (90). They also suggest that, despite the failure of eritoran in the ACCESS trial, new therapeutic potentials might still emerge for this agent.

## Toll-Like Receptor 2

Toll-like receptor 2 has been implicated in the recognition of an amazingly broad spectrum of microbial ligands originating from bacteria, fungi, viruses, and parasites (91). This property is at least partly due to the fact that TLR2 forms heterodimers with TLR1, TLR6, and possibly TLR10 (9, 15, 92). The biological relevance of TLR2 homodimers is controversial. Indeed, some ligands have been reported to trigger cells through TLR2 independently of TLR1 and TLR6. Yet, only TLR2/TLR1 and TLR2/TLR6 heterodimers have been successfully crystallized (93, 94). TLR2 represents an interesting target for numerous conditions, but clinical development of TLR2-targeting drugs has been less extensive than that of TLR4.

### TLR2 antagonistic antibodies

T2.5 is a TLR2 neutralizing mouse monoclonal antibody. T2.5 blocked Pam_3_CSK_4_ lipopeptide (a TLR1/TLR2-ligand)-stimulated NF-κB nuclear translocation and MAPK phosphorylation *in vitro*. In models of Pam_3_CSK_4_-induced toxic shock and microbial challenge with a high inoculum of heat-inactivated *Bacillus subtilis*, T2.5 prevented lethal shock-like syndrome and increased survival when administered 1 h before or up to 3 h after infection (95). Furthermore, T2.5 used in combination with the 1A6 anti-TLR4/MD-2 antibody and antimicrobial therapy protected mice from sepsis caused by *S. enterica* and *E. coli* (78). Intracellular antibodies, i.e., intrabodies, have been designed to block the intracellular translocation of TLRs from the endoplasmatic reticulum to the cell surface. αT2ib is a functional anti-TLR2 scFv intrabody comprising the variable domains of the heavy and light chains of T2.5 linked together by a synthetic (Gly_4_Ser)_3_ amino acid sequence. αT2ib bound intracellularly to TLR2 and led to retention and accumulation of TLR2 inside the endoplasmatic reticulum. Adenovirus-mediated expression of αT2ib in RAW 264.7 macrophages and mouse bone marrow derived macrophages inhibited TLR2 surface expression and TLR2-ligand-driven TNF production (96). These data suggest for a therapeutic potential of T2.5 or αT2ib in microbial infections.

Many studies have attempted to elucidate the pathogenesis of acute kidney injury associated with sepsis, which involves mechanisms similar to those occurring during ischemia/reperfusion (97). DAMPs released during infection are detected through TLR2 by immune cells recruited to the ischemic tissue and/or by cells of the ischemic tissue itself, amplifying the inflammatory response and inducing injury upon reperfusion (98, 99). Blocking TLR2 under these conditions may be cytoprotective (18). OPN-305 is a humanized anti-TLR2 IgG4 monoclonal antibody [derived from OPN-301 (98) developed by Opsona Therapeutics (Dublin, Ireland)]. OPN-305 reduced TLR2-driven pro-inflammatory cytokine production through blocking of TLR2/1 and TLR2/6 mediated signaling. In a porcine model of myocardial ischemia/reperfusion injury, pretreatment with OPN-305 or administration of OPN-305 1 h after ischemia was associated with a 50% decrease in infarct size (100). Results from a first in human phase 1 trial evaluating safety, tolerability, pharmacokinetics, and pharmacodynamics of ascending doses of OPN-305 given intravenously in healthy adult subjects have just been released (101). TLR2 occupancy and inhibition of IL-6 secretion induced by heat-killed *Listeria monocytogenes* were assessed in whole blood collected up to 90 days after treatment with either the antibody or placebo. OPN-305 was well tolerated, with no significant toxicity even at the highest dose tested. Impressively, OPN-305 at doses of 0.5 and 10 mg/kg occupied 100% of TLR2 molecules expressed on monocytes collected 14 and 90 days after challenge, respectively. IL-6 release was inhibited in a parallel manner. These results suggest that treatment with OPN-305 could provide short-term protection against ischemia/reperfusion and be adjusted to confer long-lasting blockage in the case of TLR2-mediated chronic diseases (101). A phase 2 trial assessing safety, tolerability, and efficacy of OPN-305 in kidney transplant patients has been initiated (NCT01794663).

### Other TLR2 targeting strategies

New techniques are continuously implemented to facilitate the identification of therapeutic targets for adjunctive treatment in sepsis. Immunoprecipitation with systematic evolution of ligands by exponential enrichment (SELEX) was developed to screen and identify high-affinity DNA and RNA molecules that bind to TLR2 and could be used to detect other molecules influencing TLR-driven activity. A most promising candidate, AP-177, was shown to interact with TLR2, thereby obstructing ligand binding to the receptor and inhibiting TLR2-ligand-induced NF-κB activity and IL-6 and IL-8 production in THP-1 and HEK 293 cells (102). Cell-penetrating TLR2-BB peptides have been generated and shown to interfere with TLR2-ligand-induced activation of NF-κB and MAPK and cytokine production (80). Whether these compounds will undergo clinical evaluation is unknown.

## Toll-Like Receptor 3

Toll-like receptor 3 is an endosomal PRR that senses dsRNA typically produced during viral infection (103). Experimental models comparing TLR3 wild-type and TLR3 knockout mice revealed either a protective role (West Nile virus, encephalomyocarditis virus, poliovirus, coxsackievirus, murine cytomegalovirus, herpes simplex virus), a deleterious role (West Nile virus, influenza A virus, phlebovirus), or no influence (lymphocytic choriomeningitis virus, vesicular stomatitis virus, murine cytomegalovirus, reovirus) of TLR3 on anti-viral responses (104). Therefore, TLR3 agonists and antagonists might be efficient adjunctive therapies for viral infections depending on the context. In the following sections, we describe the development of synthetic dsRNA TLR3 agonists (See TLR3 Agonists) and of synthetic ssDNA TLR3 antagonists and anti-TLR3 neutralizing antibodies (See TLR3 Antagonists).

### TLR3 agonists

The dsRNA synthetic analog polyinosinic:polycytidylic acid [poly(I:C)] is a potent immunostimulant. For clinical development, poly(I:C) was stabilized with polylysine and carboxymethylcellulose (Poly-ICLC) (Hiltonol, Oncovir, Washington, DC, USA) and used to generate poly(I:C_12_U) (rintatolimod, tradename Ampligen, Hemispherx Biopharma, Philadelphia, PA, USA) by substituting an uridylic acid at a molar ratio of 12:1 in the synthesis of the polycytidylic acid strand (105). Poly(I:C) and its derivatives have been tested in several clinical trials as adjuvants for vaccines (for both infectious diseases and cancer) and complement to HAART (highly active anti-retroviral therapy) in human immunodeficiency virus (HIV) infected patients, topics that we do not discuss here.

Poly(I:C_12_U) is highly specific for TLR3 and, unlike its parental molecule poly(I:C), does not require Melanoma Differentiation-associated protein 5 (MDA5, a cytosolic PRR for viruses), for the induction of the signaling cascade leading to type I IFN production (106). Poly(I:C_12_U) has some anti-viral activity against HIV, hepatitis B virus (HBV), coxsackie B3 virus, and several flaviviruses. Results from animal models of lethal respiratory viral infection by severe acute respiratory syndrome coronavirus (SARS-coV) and Punta Toro virus highlighted the favorable impact of intranasal treatment with poly(I:C) or poly(I:C_12_U) on survival and viral loads in infected mice (107, 108). The mode of action of poly(I:C) in the respiratory tract was linked to the induction of caspase-mediated apoptosis and ROS, which are involved in the cleavage and shedding of soluble TNF receptor blocking TNF bioactivity (109). Interestingly, poly(I:C) protected against bacterial infections in the respiratory tract as well as in the central nervous system (CNS). In a mouse model of *Pseudomonas aeruginosa* pneumonia secondary to CLP, intranasal administration of poly(I:C) improved immune activation and lowered bacterial load in the lungs compared to the untreated animals (110). Corroborating results showing enhanced phagocytosis and killing of *E. coli* by microglial cells suggest that TLR3 activation is crucial for the immune response of CNS against invading pathogens (111, 112). These data support the development of TLR3 agonists as adjuvant therapies to prevent or reduce the severity of respiratory tract infections caused by viruses and possibly bacteria. In connection with that particular field, a phase 1 safety, tolerability, and pharmacokinetic trial of nasally applied poly-ICLC in human volunteers is ongoing and will explore immune activation markers. A phase 1/2 clinical trial is assessing the immunogenicity and safety of FluMist^®^(Live attenuated influenza vaccine, MedImmune, Gaithersburg, MD, USA) intranasal influenza vaccine administered with and without a poly(I:C_12_U).

### TLR3 antagonists

More recently, TLR3 antagonists have been developed taking into consideration that TLR3 over-activation by viral dsRNA may have detrimental consequences in some situations. Indeed, it has been reported that administration of poly(I:C) in mice prior to intratracheal challenge with *S. pneumoniae* impaired bacterial clearance and increased mortality. Excessive production of type I IFN was involved in this phenomenon (113). Single-stranded DNA oligonucleotides (ssDNA ODNs) efficiently competed with dsRNA for binding to TLR3, thus inhibiting cytokine production and costimulatory molecule expression by epithelial cells, PBMCs, and dendritic cells (114, 115). The efficacy of ssDNA ODNs was demonstrated in cynomolgus macaques, where intranasal injection of ssDNAs ODNs inhibited poly(I:C)-induced cytokine production in nasal secretions (115).

In addition to microbial ligands, TLR3 senses DAMPs released from injured tissue during inflammation, for example RNA from necrotic cells, promoting an excessive inflammatory response. Interestingly, administration of a TLR3 neutralizing antibody to mice reduced cecal damage induced by gut ischemia and improved survival of animals with polymicrobial sepsis when the antibody was given 6 and 24 h after CLP surgery (116). Collectively, these data demonstrate that TLR3 works as an endogenous sensor of necrosis and a regulator of the immune response, pointing to receptor modulation as a possible adjuvant therapy for sepsis.

Work remains to be done to clearly delineate the precise role of TLR3 in viral and bacterial infections and to appraise the benefit afforded by TLR3 agonistic or antagonistic strategies for infectious diseases, especially septic shock.

## Toll-Like Receptor 5

A single TLR5 agonist has had clinical development, namely CBLB502. Flagellin is the only ligand of TLR5 described to date. Detailed structural basis of flagellin recognition by TLR5 has been obtained through crystallographic analyses, unraveling a unique mode of interaction between the two molecules as depicted from stoichiometry, ligand arrangement, and binding interfaces (117). The role of structural constraints for induction of the NF-κB signaling cascade downstream TLR5 was supported by structure-guided mutagenesis and deletion analyses on CBLB502 (Entolimob), a therapeutic agent derivative of *S. enterica* flagellin implemented by Cleveland Biolabs (Buffalo, NY, USA). CBLB502 is currently tested in a phase 1 trial in late stage cancer patients (NCT01527136). Several clinical trials have investigated the safety and adjuvant efficacy of recombinant flagellin in a number of vaccine settings (against influenza virus, *Helicobacter pylori, Campylobacter, Yersinia pestis*, West Nile virus, etc) (118).

CBLB502 plays a protective role against radiation-induced tissue injury, probably by suppressing apoptosis, attenuating ROS generation, and promoting tissue regeneration (119). These properties could explain the beneficial effect of this agonist in a murine model of acute ischemic renal failure when administered 30 min after reperfusion (120). Highlighting the favorable role of flagellin against tissue damage, results from two mouse studies suggested that protection and repair of the intestinal mucosa that serves as a first line defense barrier is the key mode of action of flagellin. In the first study, flagellin was shown to induce the expression of RegIIIγ, a C-type lectin with bactericidal activity, and to restrict small intestine colonization with vancomycin-resistant *Enterococcus* (VRE) in animals inoculated with VRE via oral gavage (121). In the second study, treatment with flagellin reduced intestinal epithelium destruction induced by dextran sodium sulfate (a chemical used to induce severe acute colitis) and increased survival of mice inoculated with *S. typhimurium* by oral gavage (122). These data suggest that flagellin or TLR5 agonists may represent attractive tools for treating pathologies that injure the intestinal tract, including severe sepsis. No TLR5 antagonists have been reported.

## Toll-Like Receptor 9

### TLR9 agonists

Toll-like receptor 9 agonists tested in clinical trials are synthetic CpG oligodeoxynucleotides (CpG ODNs) among which CPG10101, IMO-2125, SD-101, and CpG 7909 that mimic unmethylated CpG dinucleotide-rich sequences enriched in microbial DNA. CpG ODNs are powerful immunostimulants, exploited for their adjuvant properties in vaccines against infectious diseases (flu, malaria, HIV infection, pneumococcal and meningococcal diseases) and cancer (melanoma, leukemia, glioblastoma, and colorectal, prostate and breast cancer) (123). The adjuvant properties of CpG ODNs have been used to enhance the phagocytosis and the killing of bacteria (*S. typhimurium* and *S. pneumoniae*) by phagocytic cells (124, 125). Interestingly, a recent study showed that CpG ODN given 1 h prior to CLP surgery prevented CLP-induced cardiac dysfunction in mice. The authors proposed that targeting TLR9 could be a useful approach for the management of cardiovascular dysfunction in severe sepsis patients (126).

Therapeutic strategies focusing on TLR9-mediated immunomodulation are currently being implemented for chronic viral infections, such as chronic hepatitis C (HCV). Plasmacytoid dendritic cells are the main cells producing type I IFN and are therefore considered to play an important role in viral infections. TLR9 agonists stimulate plasmacytoid dendritic cells to produce large amounts of type I IFN, especially IFNα, which is the backbone of therapy for HCV. Indeed, IFNα powerfully inhibits viral replication and promotes innate and adaptive host immune responses. Moreover, IFN production appears to be impaired in plasmacytoid dendritic cells of HCV patients (127, 128).

CPG10101 was originally developed under the trade name Actilon by Coley Pharmaceuticals (Wellesley, MA, USA), a company recently incorporated by Pfizer (New York City, NY, USA). CPG10101 has undergone two phase 1 studies with promising results. A phase 1a study for drug safety and pharmacokinetics conducted in 48 healthy volunteers revealed well tolerated immunostimulatory effects without serious adverse events even when using high doses of CPG10101 (129). CPG10101 was also tested in 60 HCV patients, 50 infected with genotype 1 HCV. CPG10101 was administered subcutaneously to four randomized groups at different doses twice per week for 4 weeks alone or in combination with pegylated IFNα and ribavirin. The TLR9 agonistic effect of CPG10101 was associated with the induction of IFNγ and IFNα and the decrease of viral loads. The only serious adverse events were urticaria and pruritis, without manifestation of respiratory complications (130). A phase 2 study enrolling 113 non-responders genotype 1 HCV patients has been completed, but results have not been released yet.

IMO-2125, manufactured by Idera Pharmaceuticals (Cambridge, MA, USA), has undergone two phase 1 trials: one for dose estimation and one for safety, pharmacokinetics, and pharmacodynamics, enrolling 60 and 40 treatment-naïve genotype 1 HCV patients, respectively. IMO-2125 administration dose-dependently decreased viral loads, increased the production of anti-viral cytokines and chemokines especially IFNα, and activated NK and T-cell responses (41, 42). A phase 2 trial was planned, but in April 2011 the company postponed its initiation. The decision was made based on histological data from a 26-week non-clinical toxicology study of IMO-2125 in rodents and non-human primates^4^. Preliminary analyses suggested evidence of atypical lymphocytic proliferation, although no adverse events were reported in humans. Thorough analysis results are pending.

A phase 1b study sponsored by Dynavax (Berkeley, CA, USA) investigated the safety and efficacy of SD-101 in chronic HCV. SD-101 was administered as monotherapy or in combination with ribavirin to 34 chronically infected, treatment-naïve, genotype 1 HCV patients. Results released in 2010 indicated that SD-101 was well tolerated and safe without any serious adverse events. The drug had significant anti-viral activity based on dose-dependent anti-viral response, with 100% of patients at the highest dose showing more than one log reduction in viral load, and increased expression of type I IFN-dependent anti-viral genes (IP-10, MCP-1 MX-B, ISG-54K). These data comfort results from *in vitro* studies showing that SD-101 stimulated human PBMCs to produce 20-fold higher levels of both IFNα and IFNλ in comparison with first-generation TLR9 agonists (40).

### TLR9 antagonists

Bacterial DNA released during infection is a MAMP, and exuberant activation of TLR9 may participate to the sepsis pathophysiology. Hence, drug inhibitors of TLR9 may have therapeutic potential in human sepsis. As a proof of concept, administration up to 12 h after surgery of a single dose of an inhibitory CpG ODN blocking TLR9 signaling protected mice from polymicrobial sepsis following CLP (131). HMGB proteins are essential for triggering nucleic acid receptor-mediated innate immune responses. HMGB-binding non-immunogenic-ODNs have been designed to inhibit HMGB-mediated pathologies. A non-immunogenic ODN termed ISM ODN was tested in a mouse model of endotoxemia. Impressively, 70% of mice treated with ISM ODN survived up to 72 h after LPS challenge, while all mice from the control group died within 48 h (132). These data argue for a possible use of non-immunogenic-ODNs in therapeutic interventions. Antimalarial drugs such as chloroquine are well known for their anti-inflammatory properties in autoimmune diseases such as rheumatoid arthritis and systemic lupus erythematosus (133). Chloroquine blunted cytokine production and protected mice from toxic shock induced by CpG ODN and LPS. Chloroquine down-regulated the expression of both TLR9 and TLR4, suggesting that it acts at multiple levels to inhibit inflammation (134, 135). Additionally, when administered 6 h after CLP in elderly mice treated with fluids and antimicrobials, chloroquine significantly improved survival, strengthened renal function and protected from multiple organ dysfunction (136). These results support clinical evaluation of chloroquine in patients with severe sepsis, especially those presenting with acute renal failure. Based on its anti-inflammatory activity and because it inhibits endosomal acidification which are important for cell infection by viruses, chloroquine is also tested in multiple trials for prevention and/or treatment of viral infections (HIV, influenza, dengue, chikungunya). Yet, the mode of action of chloroquine is multi-factorial and not only through TLR9.

## Toll-Like Receptor 7 and 8

Toll-like receptor 7 and 8 are closely related TLRs well known for their capacity to recognize ssRNA from viruses such as HCV, HBV, HIV, influenza virus, Herpes simplex virus, Epstein-Barr virus, vesicular stomatitis virus, papilloma virus, respiratory syncytial virus, and Sendai virus. In agreement, TLR7 is primarily expressed in plasmacytoid dendritic cells. More recently, TLR7 and TLR8 were shown to sense bacterial RNA released within phagosomal vacuoles (137). TLR7 and TLR8 triggering induces potent anti-viral immune responses characterized by the production of type I IFNs and NF-κB-dependent cytokines. TLR7/8 agonists are primarily developed for treating viral diseases, but also as adjuvants for cancer and infectious disease vaccines.

### TLR7/8 agonists in chronic viral infections

Imiquimod (Aldara, originally developed by 3M Pharmaceuticals, Maplewood, MN, USA) is the only TLR7 agonist marketed for anti-viral treatment, i.e., external ano-genital warts caused by human papilloma virus. Numerous TLR7/8 agonists are in clinical development, like CL097, isatoribine, ANA975, ANA773, PF-04878691 (852A), R-848 (resiquimod), and GS-9620.

Although therapeutic strategies for HCV have evolved in the recent years, quest of new immunomodulatory targets remains mandatory. Treatment with new agents such as protease inhibitors appears to be efficient but presents with issues of resistance in the long run (138). Moreover, current protocol therapy with IFNα provides replenishment with a specific subtype of this cytokine. However, use of TLR7 agonists is able to induce a variety of IFN subtypes, possibly providing a more radical and integrated anti-viral activity (139). Finally, administration of TLR7/8 agonists may overcome the adverse events caused by IFNα, like the suppression of granulocyte colony stimulating factor (G-CSF) leading to neutropenia. Indeed, CL097 reversed IFNα-mediated inhibition of G-CSF production by PBMCs obtained from HCV patients and healthy volunteers (140). CL097 also restored defective cytokine production by myeloid dendritic cells from HIV patients (141).

Isatoribine (Anadys Pharmaceuticals, San Diego, CA, USA) was one of the first guanosin analog selective TLR7 agonists to be implemented and tested on humans (142). Intravenous administration of isatoribine to 12 HCV patients during a 7-day treatment plan resulted in reduced plasma concentration of HCV RNA, regardless virus genotype. Adverse events comprised dose-dependent joint pain, decreased white blood cells, and platelets counts, insomnia, and headache (143). The development of an oral prodrug that could lack the detrimental effects of isatoribine, especially in the gastrointestinal tract, pointed to a new candidate, ANA975. Preliminary results from a study conducted with ANA975 were promising. Oral administration of ANA975 presented with elevated plasma levels of isatoribine at a concentration able to reduce HCV RNA in the plasma of infected patients (144). Unfortunately, Anadys Pharmaceuticals and Novartis (Basel, Switzerland) announced in 2007 discontinuation of drug development due to unacceptable toxicity in pre-clinical animal studies (145).

Subsequent elaboration of an oral prodrug of isatoribine by Anadys Pharmaceuticals led to the generation of ANA773. ANA773 exhibits efficient induction of endogenous type I IFNs. A double-blind, placebo-controlled study was conducted in 34 patients with chronic HCV, either treatment-naïve or relapsed from IFN-based therapy. Interestingly, ANA773 was safe and well tolerated and presented only with grade 1 and 2 adverse events. Moreover, ANA773 dose-dependently decreased HCV RNA levels (48). In another study, repeated treatment with ANA773 was associated with transient decrease of myeloid and plasmacytoid dendritic cells and increased levels of IFNα and IP-10 in the blood of patients achieving a reduction in the viral load, suggesting an impairment in IFNα production in the case of non-responders (49).

PF-04878691, formerly known as 852A, is a TLR7 agonist generated by Pfizer and implemented so that repeated low doses of the drug would be accompanied with the benefits of the agonistic activity without adverse events. A proof of concept study was conducted to evaluate safety and tolerability of the drug and to determine pharmacokinetics and pharmacodynamics. Twenty-four healthy volunteers received orally increasing doses of PF-04878691. PF-04878691 induced immune response in a dose-dependent and frequency-related manner. However, two of the subjects exhibited severe lymphopenia along with flu-like symptoms and hypotension (50). In an attempt to decide on the future perspectives of this compound, a model was used to predict the safety and efficacy of PF-04878691 in HCV patients. This model exploited clinical results from the former study in healthy volunteers along with those reported from the use of CPG10101. Further optimization will be required before entering the drug in a phase 2 study (146).

Other TLR7/8 agonists have reached phase 3 trials, but demonstrated lack of efficacy and serious adverse effects. When monocytes from HIV patients were stimulated with resiquimod, IL-12 secretion was augmented while TNF production was decreased compared to the control group. Additionally, HIV replication in cultured monocytes was inhibited (147). These promising *in vitro* results were reproduced in a phase 2 trial that enrolled patients with herpes simplex virus type 2. Topical application of resiquimod protected from viral lesion spreading (148). However, a phase 3 trial disclosed lack of efficacy of the drug and, although a phase 2 study for the treatment of HCV demonstrated decreased viral loads, adverse side effects similar to those resulting from IFNα treatment were of serious concern (149).

Two phase 1 clinical trials for the safety and pharmacokinetics of a novel compound, GS-9620 (Gilead Sciences, Foster City, CA, USA), are currently enrolling treatment-naïve and viral suppressed HBV patients respectively, while another one enrolling HCV patients has been recently approved. GS-9620 is a TLR7 agonist tested originally in HBV infected chimpanzees. Drug administration was associated with reduced viral loads both in plasma and liver, probably through enhanced apoptosis of hepatocytes (150). Testing of ascending dosages of the drug in healthy volunteers presented with flu-like adverse events at a dose of 8–12 mg, but cytokine induction was achieved even at administration of 2 mg pointing to a promising adjunctive treatment for chronic viral infections (151).

### TLR7/8 agonists in acute bacterial and viral infections

All the above data illustrate the efforts devoted to the development of TLR7/8 agonists for treating viral pathologies. Taking into account that bacterial RNA triggers TLR7 and TLR8 pathways, one may speculate that TLR7/8 agonists would impact on bacterial sepsis. Unfortunately, there are almost no data available on that subject. In one report, intravenous injection of R-848 prior to sepsis induction using the colon ascendens stent peritonitis model increased cytokine release and bacterial clearance, but the effect on survival was not reported (152). Finally, considering that excessive TLR activation induced by viruses or bacteria may have detrimental effects for the host, it might be of interest to generate TLR7/8 antagonists to counteract overwhelming immune activation in conditions of severe infections.

## Conclusion

Targeting TLRs is a promising field for sepsis management and infection control. TLR agonists are widely used to optimize vaccine efficacy, taking advantage of their powerful adjuvancity. Whether TLR agonists and antagonists will have such success for treatment therapies, especially for sepsis, has to be established. Agonists of TLR3 and TLR7-9 yielded very promising results for treating viral infections. Only few studies tested antagonistic drugs. Mainly for historical reasons and because of their ligand specificity, TLR4 and to a lesser extent TLR2 are the favorite targets for developing anti-sepsis drugs. This domain of research has monopolized important resources, but unfortunately many drugs tested in animal models have not entered human studies. Those that proceeded, like eritoran and TAK-242, did not achieve primary endpoint goal and/or were accompanied with manifestation of adverse events. Animal models provide invaluable information about the role of TLRs and the mechanism underlying infection pathogenesis, but lack complexity in terms of comorbidities and host response compared to human disease (153). So, how to explore more efficiently new treatment strategies? Improving the design of clinical studies is mandatory. We should discriminate those patients that could benefit from therapy based on their genotype (for example affecting TLR pathways) and the expression of the targeted molecule, and select homogeneous, well-described population bearing the same underlying conditions and disease severity (68–70, 154). Ideally, sepsis studies should use specific biomarkers to help patient enrollment and weigh treatment efficacy in realistic conditions (155). The failure of the recent trials in patients with severe sepsis has taught us valuable lessons regarding patient selection, time of intervention, and follow-up (68–70). Blocking one single mediator may also not be sufficient to interfere with sepsis. Developing sepsis-specific TLR-targeted therapies for patients is a path strewn with obstacles, but an exciting and promising area of research. These studies offer new possibilities for prevention and intervention in infectious diseases, but also for other conditions, such as cancer, allergy, asthma and autoimmune diseases either directly or through improvement of vaccines.

## Conflict of Interest Statement

The authors declare that the research was conducted in the absence of any commercial or financial relationships that could be construed as a potential conflict of interest.

## Footnotes

^1^http://www.survivingsepsis.org

^2^www.clinicaltrials.gov

^3^http://www.novimmune.com

^4^http://ir.iderapharma.com
